# Retrospective Study of 43 Cases of Visceral Artery Aneurysms: Evaluation of Surgical Indications Based on the 2020 Revised Guidelines of the Society for Vascular Surgery

**DOI:** 10.3400/avd.oa.24-00128

**Published:** 2025-06-13

**Authors:** Nozomu Ishikawa, Kazunori Inuzuka, Masaki Sano, Kazuto Katahashi, Hajime Tsuyuki, Yusuke Endo, Takaaki Saito, Hiroya Takeuchi, Naoki Unno

**Affiliations:** 1 Division of Vascular Surgery, Hamamatsu University School of Medicine, Hamamatsu, Shizuoka, Japan; 2 Second Department of Surgery, Hamamatsu University School of Medicine, Hamamatsu, Shizuoka, Japan; 3 Department of Vascular Surgery, Iwata City Hospital, Iwata, Shizuoka, Japan; 4 Department of Vascular Surgery, Hamamatsu Medical Center, Hamamatsu, Shizuoka, Japan

**Keywords:** renal artery aneurysm, splenic artery aneurysm, hepatic artery aneurysm, pancreaticoduodenal artery aneurysm, gastroepiploic artery aneurysm

## Abstract

**Objectives:** Advancements in imaging technology have led to an increase in the incidental detection of visceral artery aneurysms (VAAs), which are associated with high mortality when ruptured. In 2020, the Society for Vascular Surgery (SVS) released updated guidelines, replacing the previous 2005 ACC/AHA recommendations. This study aimed to evaluate the impact of the new guidelines through a retrospective analysis of VAA cases treated at our department.

**Methods:** We retrospectively reviewed 43 cases of VAA treated between 2002 and 2024 at our department. Each case was re-evaluated to determine whether it met the treatment criteria defined in the 2020 SVS guidelines.

**Results:** Of the 43 cases, 23 (53.5%) met the new guideline criteria. Notably, treatment eligibility for renal and splenic artery aneurysms decreased significantly due to the revised aneurysm size threshold, raised from 2 to 3 cm. The remaining 20 cases were considered ineligible based solely on size, with the exception of cases involving young female patients, ruptured aneurysms, symptomatic lesions, or pseudoaneurysms.

**Conclusions:** The 2020 SVS guidelines impose stricter treatment indications for VAAs. However, clinical decisions should also consider aneurysm location, patient age, gender, symptoms, and rupture risk on an individual basis.

## Introduction

Visceral artery aneurysms (VAAs) are aneurysms that occur in the celiac artery, superior mesenteric artery, renal artery, inferior mesenteric artery, and their branches. With recent advancements in imaging technology, the incidental detection of VAAs has increased. Ruptured VAAs can lead to life-threatening conditions, and the mortality rate following treatment intervention varies between 15.5% and 70%, depending on the year of the report and the location of the aneurysm.^1)^ Therefore, accurate diagnosis and appropriate treatment prior to rupture are of paramount importance.

Two major guidelines address the management of VAAs: the European Society for Vascular Surgery (ESVS) guidelines on mesenteric artery aneurysms published in 2017,^2)^ and the Society for Vascular Surgery (SVS) guidelines on VAAs issued in 2020.^3)^ Notably, the latter specifies aneurysm size thresholds for surgical indications based on the anatomical location of the VAA.

In this study, we retrospectively analyzed VAA cases that underwent treatment intervention at our department. We compared the treatment indications and procedural choices with the criteria outlined in the 2020 SVS guidelines, assessing the impact of aneurysm size on treatment decisions.

## Materials and Methods

We retrospectively analyzed cases of VAAs that underwent treatment intervention at our department between January 2002 and March 2024. We reviewed patient backgrounds, treatment indications, procedures, and postoperative complications. Postoperative renal dysfunction was defined according to the Kidney Disease: Improving Global Outcomes (KDIGO) guidelines as an increase in serum creatinine of ≥0.3 mg/dL compared to preoperative levels.^4)^

Before the publication of the SVS guidelines, the treatment indications for VAA at our department were based on the 2005 American College of Cardiology/American Heart Association (ACC/AHA) guidelines.^5)^ Treatment intervention was indicated for any symptomatic aneurysms or pseudoaneurysms, aneurysms with a diameter ≥2 cm, those with rapid enlargement over a short period, and splenic artery aneurysms (SAAs) in pregnant or potentially pregnant women, as well as aneurysms in the gastroduodenal arcade, regardless of size.

Regarding the choice of procedure, endovascular therapy (EVT) such as coil embolization was the 1st-line treatment when peripheral organ blood flow was expected to be maintained even after the closure of the inflow/outflow arteries. When EVT was not feasible, open surgery (OS), such as simple ligation or aneurysm resection, was chosen as the 2nd-line treatment. In cases where EVT posed a risk of insufficient peripheral organ perfusion, a stent graft was considered, and if this was not possible, OS with vascular reconstruction was selected.

The 2020 SVS guidelines specify treatment indications based on aneurysm size for each VAA location (**Table 1**). The size thresholds for treatment intervention were set at 3 cm for renal artery aneurysms (RAAs) and SAAs, and 2 cm for celiac artery aneurysms (CAAs), hepatic artery aneurysms (HAAs), and jejunal/ileal artery aneurysms. For other locations, treatment was indicated regardless of aneurysm size.

**Table:** Table 1 Surgical indications in the 2020 SVS guidelines

Aneurysm	Surgical indications
RAA	>3 cm
	Any size: symptomatic cases, ruptured aneurysms, women of childbearing potential, and cases with refractory hypertension due to renal artery stenosis.
SAA	≥3 cm
	Any size: ruptured cases, symptomatic cases, pseudoaneurysms, increase in size, and women of childbearing potential.
CAA	>2 cm
	Any size: ruptured cases, symptomatic cases, pseudoaneurysms, and increase in size.
Gastric artery aneurysm and GEAA	Any size (treatment indication applies regardless of aneurysm size or the presence of symptoms)
HAA	>2 cm
	Any size: symptomatic cases, pseudoaneurysms, enlargement of 0.5 cm/year, vasculopathy or vasculitis, and positive blood cultures.
SMAA	Any size (excluding dissections)
Jejunal and ileal artery aneurysms	>2 cm
	Any size: ruptured cases, symptomatic cases, and pseudoaneurysms.
Colic artery aneurysm	Any size (treatment indication applies regardless of aneurysm size or the presence of symptoms)
PDAA and GDAA	Any size (treatment indication applies regardless of aneurysm size or the presence of symptoms)

SVS: Society for Vascular Surgery; RAA: renal artery aneurysm; SAA: splenic artery aneurysm; CAA: celiac artery aneurysm; GEAA: gastroepiploic artery aneurysm; HAA: hepatic artery aneurysm; SMAA: superior mesenteric artery aneurysm; PDAA: pancreaticoduodenal artery aneurysm; GDAA: gastroduodenal artery aneurysms

This study was approved by the Ethics Review Committee of Hamamatsu University School of Medicine (Approval No. 17-129).

## Results

During the study period, 40 patients underwent treatment for a total of 43 VAAs. The patient background showed a nearly equal gender ratio (male-to-female ratio of approximately 1:1), and 57.5% of patients had hypertension (**Table 2**). No connective tissue disease was observed in any of the cases.

**Table:** Table 2 Patient characteristics and backgrounds in visceral artery aneurysms

Male sex, n	21 (52.5%)
Age, years	61.6 ± 11.9
Body mass index, kg/m^2^	22.62 (20.19–24.82)
Preoperative eGFR, mL/min/1.73m^2^	73.86 ± 24.16
Smoking, n	14 (35.0%)
Hypertension, n	23 (57.5%)
Dyslipidemia, n	18 (45.0%)
Diabetes mellitus, n	6 (15.0%)
COPD, n	3 (7.5%)
Coronary artery disease, n	3 (7.5%)

Age and preoperative eGFR are presented as mean ± standard deviation. BMI is presented as median (interquartile range). The preoperative eGFR data is for n = 36. eGFR: estimated glomerular filtration rate; COPD: chronic obstructive pulmonary disease

The anatomical distribution of the VAAs was as follows: 11 RAAs (7 on the left, 4 on the right), 13 SAAs, 2 CAAs, 3 gastric and gastroepiploic artery aneurysms (GEAAs), 5 HAAs (3 common HAAs [CHAAs] and 2 proper HAAs [PHAAs]), 3 superior mesenteric artery aneurysms (SMAAs), 1 inferior mesenteric artery aneurysm (IMAA), and 5 pancreaticoduodenal artery aneurysms (PDAAs).

The characteristics of each VAA are summarized in **Table 3**. Aneurysms with a median size of less than 2 cm were observed in GEAA, SMAA, and PDAA cases. All RAAs were saccular aneurysms, and overall, 83.7% of the aneurysms were saccular. Three aneurysms were dissecting, with 1 case in the SAA and 2 cases in the SMAA. Rupture was observed in 2 cases of PDAA and 1 case of SAA, all of which were treated with emergency coil embolization. Two patients underwent treatment for multiple VAAs. One patient had coexisting CAA and PDAA, both treated simultaneously with OS. Another patient had both CHAA and SAA, which were treated simultaneously with coil embolization; however, 4.5 years later, the SMAA enlarged, and OS was performed. In 7 patients, additional VAAs that did not meet treatment criteria were observed. Seven patients presented with compression of the celiac artery origin due to median arcuate ligament syndrome (MALS), which was associated with the development of collateral flow through the pancreaticoduodenal arcade and the presence of 1 HAA, 1 IMAA, and 5 PDAAs. Regarding the treatment methods, OS was performed in 25 VAAs, while EVT was used in 18 VAAs. EVT was more commonly performed for SAAs, whereas OS was more frequently chosen for other aneurysm locations (**Table 4**).

**Table:** Table 3 The characteristics of visceral artery aneurysms

Aneurysm	N (male:female)	Median size (mm)(range)	Saccular (%)	Dissection (%)	Calcification (%)	Rupture (%)
RAA	11 (4:7)	20.0 (15–35)	11 (100)	0 (0.0)	2 (15.4)	0 (0.0)
SAA	13 (6:7)	24.0 (9.0–42)	12 (92.3)	1 (7.7)	9 (69.2)	1 (7.7)
CAA	2 (1:1)	20.0 (20–20)	1 (50)	0 (0.0)	0 (0.0)	0 (0.0)
GEAA	3 (3:0)	12.3 (6.0–28)	3 (100)	0 (0.0)	0 (0.0)	0 (0.0)
HAA	5 (3:2)	22.0 (17–26)	4 (80)	0 (0.0)	0 (0.0)	0 (0.0)
SMAA	3 (2:1)	16.0 (15–35)	1 (33.3)	2 (66.7)	0 (0.0)	0 (0.0)
IMAA	1 (0:1)	49	1 (100)	0 (0.0)	1 (100)	0 (0.0)
PDAA	5 (3:2)	18.0 (3.0–25)	3 (60)	0 (0.0)	1 (20.0)	2 (40.0)
TOTAL	43	20.0 (3.0–49)	36 (83.7)	3 (7.0)	13 (30.2)	3 (7.0)

RAA: renal artery aneurysm; SAA: splenic artery aneurysm; CAA: celiac artery aneurysm; GEAA: gastroepiploic artery aneurysm; HAA: hepatic artery aneurysm; SMAA: superior mesenteric artery aneurysm; IMAA: inferior mesenteric artery aneurysm; PDAA: pancreaticoduodenal artery aneurysm

**Table:** Table 4 Treatment procedures for visceral artery aneurysms by location

Aneurysm	OS (n = 25)	EVT (n = 18)	
RAA	10	1	Ex vivo repair with autotransplantation (10), coil embolization (1)
SAA	1	12	Coil embolization (12), laparoscopic splenectomy (1)
CAA	2	0	Aneurysmectomy and GSV patch plasty (1), ligation and GSV bypass (1)
GEAA	2	1	Coil embolization (1), laparoscopic aneurysmectomy (1), aneurysmectomy with distal gastrectomy (1)
HAA	3	2	Aneurysmectomy and autogenous vein bypass (3), coil embolization (2)
SMAA	3	0	Aneurysmectomy and GSV repair (2), aneurysmorrhaphy (1)
IMAA	1	0	Aneurysmectomy and direct anastomosis (1)
PDAA	3	2	Coil embolization (2), aneurysmectomy and GSV bypass (2), ligation (1)

OS: open surgery; EVT: endovascular therapy; RAA: renal artery aneurysm; SAA: splenic artery aneurysm; CAA: celiac artery aneurysm; GSV: great saphenous vein; GEAA: gastroepiploic artery aneurysm; HAA: hepatic artery aneurysm; SMAA: superior mesenteric artery aneurysm; IMAA: inferior mesenteric artery aneurysm; PDAA: pancreaticoduodenal artery aneurysm

### Follow-up after treatment

The 2020 SVS guidelines recommend follow-up intervals of 1 year or every 1–2 years. However, at our institution, we have traditionally conducted follow-ups every 6 months in outpatient settings. As for imaging studies, we alternate between non-contrast computed tomography (CT) and ultrasound every 6 months. While non-contrast CT is the standard, contrast-enhanced CT is also used in cases where aneurysm visualization is difficult, provided there are no renal function concerns. This approach allows us to monitor aneurysm size progression and detect any residual blood flow within the aneurysm in cases of coil embolization.

### RAA (total 11 cases)

Of the 11 RAAs, all cases were located at the renal hilum. Among these, 10 cases were treated with autotransplantation and ex vivo reconstruction.^6)^ One case, which had a history of Y-graft replacement for a ruptured abdominal aortic aneurysm, was treated with coil embolization.

According to the 2020 SVS guidelines, treatment indications for RAAs with simple morphology are limited to aneurysms with a diameter of 3 cm or more. In this study, only one of the RAA at the main renal artery met this criterion.

Therefore, based on the 2020 SVS guidelines, the number of surgical cases would decrease from 11 to 1.

### SAA (total 13 cases)

Of the 13 SAAs, 12 cases—including 1 ruptured aneurysm and 1 infectious aneurysm due to a retroperitoneal abscess following total gastrectomy—were treated with coil embolization. One aneurysm located at the splenic hilum was judged to have a high risk of extensive splenic infarction from embolization, and as a result, laparoscopic splenectomy was performed. Three aneurysms had a diameter of 3 cm or larger. According to the 2020 SVS guidelines, 5 aneurysms, including the 2 symptomatic ones, met the criteria for treatment intervention.

### CAA (total 2 cases)

In both cases of CAAs, autologous vein revascularization was performed. In 1 case, the aneurysm wall was resected, and then a patch was created using the great saphenous vein (GSV). In the other case, the inflow and outflow arteries were ligated, the aneurysm was left in place, and a right common iliac artery to proper hepatic artery bypass was created using the GSV. The coexisting PDAA in this case was treated by ligating both inflow and outflow arteries.

According to the 2020 SVS guidelines, aneurysms with a diameter of 2 cm or larger are indicated for treatment intervention. In our cases, both aneurysm diameters were 2 cm, meeting the 2020 SVS guidelines for treatment intervention.

### GEAA (total 3 cases)

In 1 case of gastric artery aneurysm associated with gastric cancer, GEAA resection was performed during open gastrectomy. For the other 2 cases, coil embolization and laparoscopic aneurysm resection were performed, respectively. The choice of procedure was based on the patients’ general condition, comorbidities, and vascular anatomy. According to the 2020 SVS guidelines, all gastric and GEAAs, regardless of size, are recommended for treatment (recommendation grade 1-B). All procedures in our cases were performed in accordance with these guidelines.

### HAA (total 5 cases)

In 2 cases of CHAAs, coil embolization was performed after imaging confirmed that hepatic blood flow was maintained through the pancreaticoduodenal arcade. For 1 CHAA extending distally to the proper hepatic artery/gastroduodenal artery bifurcation and 2 PHAAs, aneurysm resection and autologous vein revascularization were performed. According to the 2020 SVS guidelines, aneurysms with a diameter of 2 cm or larger qualify for treatment intervention, and in our study, 3 cases met this criterion. In 1 case, coil embolization was also performed for a concomitant SAA, which required treatment. Another case involved a 19-mm saccular aneurysm, which was treated preemptively due to the likelihood of requiring surgery in the near future. Therefore, under the 2020 SVS guidelines, the number of treatment cases decreases from 5 to 3.

### PDAA (total 5 cases)

In 1 case, the inflow and outflow arteries were ligated during an OS performed simultaneously with the CAA. Two cases underwent aneurysm resection and bypass surgery using the GSV, with 1 of these cases involving an OS performed concurrently with a hepatectomy for hepatocellular carcinoma. Coil embolization was performed in 2 cases, both of which were ruptured aneurysms. In 1 case, due to MALS, the blood flow in the gastroduodenal artery was retrograde. Laparoscopic median arcuate ligament release was performed 1.5 months after the coil embolization, and the blood flow subsequently became antegrade. In the other case, MALS was also present but with only mild stenosis. After coil embolization of the aneurysm, the patient has been under outpatient follow-up for MALS. According to the 2020 SVS guidelines, PDAAs should be treated regardless of size to prevent rupture, and all treatments in this study followed these guidelines.

### SMAA (total 3 cases)

In all 3 cases of SMAAs, the risk of intestinal ischemia due to embolization was deemed high. Therefore, revascularization using the GSV was performed in 2 cases via OS, and aneurysm plication was performed in the other case. According to the 2020 SVS guidelines, all SMAAs should be treated regardless of size (recommendation grade 1-A), and for dissecting SMAAs, careful observation is recommended in the absence of intractable pain (recommendation grade 2-B). In our cases, 1 SMAA was dissecting, but due to the aneurysm’s tendency to enlarge and its size of 35 mm, surgery was performed.

Thus, following the 2020 SVS guidelines, all procedures in our cases were performed in accordance with these guidelines.

### IMAA (total 1 case)

This case involved MALS with celiac artery origin compression and SMA root occlusion, which led to a high risk of intestinal ischemia. Aneurysm resection and end-to-end anastomosis were performed. There are no specific guidelines for IMAA, but if it is classified as a type of colonic artery aneurysm, the 2020 SVS guidelines recommend treating all colonic artery aneurysms (recommendation grade 1-B). Thus, according to the 2020 SVS guidelines, this case was deemed eligible for surgery.

### Postoperative complications

During a median postoperative follow-up period of 101 months (with a range of 2–270 months), no recurrence or rupture of VAAs was observed. In the early postoperative period, serum creatinine data were unavailable for 5 patients who had undergone OS for 6 VAAs (1 RAA, 1 CAA, 1 HAA, 1 SMAA, and 2 PDAAs). Additionally, 1 SMAA case treated with aneurysmorrhaphy was a dialysis patient, and thus postoperative renal function was evaluated in 34 patients with 36 VAAs (OS: 19, EVT: 16). Transient postoperative renal dysfunction was observed in 4 cases following autotransplantation and ex vivo reconstruction for RAA. Partial splenic infarction was noted in 4 cases after coil embolization for SAA. One patient who underwent simultaneous surgery for CAA and PDAA, and another who had revascularization with an autologous vein for SMAA (with a history of open gastrectomy), developed adhesive small bowel obstruction in the late postoperative period (10 years after surgery), requiring inpatient conservative treatment. One patient who underwent aneurysm resection and revascularization with an autologous vein for PHAA experienced transient postoperative renal dysfunction. None of the cases required invasive additional treatments or renal replacement therapy postoperatively.

In this study, we compared the treatment indications for 43 VAAs treated at our department with those outlined in the SVS guidelines. As a result, 23 aneurysms (53.5%) met the criteria for surgical intervention (**Table 5**). Specifically, only 1 out of 11 RAAs (9.1%) and 5 out of 13 SAAs (38.5%) were eligible for treatment according to the guidelines. Additionally, other aneurysms that did not meet the treatment criteria outlined in the guidelines included 2 CHAAs.

**Table:** Table 5 Comparison of the number of treatment cases and SVS guideline-eligible cases for 43 visceral artery aneurysms treated at our department

Aneurysm	Treatment cases	SVS guideline-eligible cases
RAA	11	1
SAA	13	5
CAA	2	2
GEAA	3	3
HAA	5	3
SMAA	3	3
IMAA	1	1
PDAA	5	5
TOTAL	43	23

SVS: Society for Vascular Surgery; RAA: renal artery aneurysm; SAA: splenic artery aneurysm; CAA: celiac artery aneurysm; GEAA: gastroepiploic artery aneurysm; HAA: hepatic artery aneurysm; SMAA: superior mesenteric artery aneurysm; IMAA: inferior mesenteric artery aneurysm; PDAA: pancreaticoduodenal artery aneurysm

## Discussion

In recent years, advancements in imaging technology have led to an increase in the incidental detection of unruptured VAAs.^7–9)^ Multi-detector CT allows for 3-dimensional reconstruction, providing more detailed information on aneurysm morphology, and the course of the inflow and outflow arteries, which contributes significantly to the selection of the surgical approach. Additionally, for pregnant patients in whom radiation exposure should be avoided, recent improvements in MR angiography have made it a useful tool for diagnosis.^3)^

VAAs are relatively rare, and there have been no prospective, multi-center trials to establish clear guidelines for treatment based on the site of occurrence. According to the 2005 ACC/AHA guidelines, VAAs were generally deemed eligible for treatment intervention when the aneurysm diameter was 2 cm or larger,^5)^ and we followed the same criteria in our practice. In fact, the “Guidelines on Coil Embolization for Visceral Artery Aneurysms” issued by the Japanese Society of Interventional Radiology also state that the treatment indication for VAAs is a diameter of 2 cm or larger.^10)^ However, the optimal treatment approach varies depending on the characteristics and location of the aneurysm, with options including OS, laparoscopic surgery, and EVT. Standardizing treatment strategies is challenging, and both the indications for treatment and the choice of technique vary across institutions and departments.

In this context, the 2017 ESVS guidelines introduced recommendations for mesenteric arterial aneurysms. They suggest that revascularization should be preferred over arterial occlusion when feasible and when the associated risks are not high (recommendation grade 1-C).^2)^ Additionally, for anatomically appropriate cases, EVT should be considered over OS because of its association with fewer perioperative complications (recommendation grade 2a-C).^2)^

The 2020 SVS guidelines provided clearer treatment strategies for VAAs by specifying, for the 1st time, aneurysm size thresholds for treatment indications based on the anatomical location.^3)^ OS is recommended as the 1st-line treatment only for RAAs, while for other aneurysms, EVT is recommended as the 1st choice if anatomically feasible. In Japan, the use of stent grafts in elective VAA surgery has not yet been approved. However, the guidelines recommend stent grafts for RAAs (recommendation grade 2-B) and for GDAAs and PDAAs (recommendation grade 2-C). Furthermore, stent grafts are explicitly recommended for HAAs if anatomical conditions are suitable. The establishment of these guidelines has provided clearer treatment strategies for VAAs than ever before. The indications for stent grafts in Japan are expected to expand in the future.

To date, there have been no retrospective studies from Japan evaluating the invasive treatment of VAAs in accordance with the SVS guidelines, as in our study. In this study, we reviewed 43 cases of VAA treated at our department based on the 2020 SVS guidelines and found that approximately half (23 cases) met the treatment criteria. These included cases of infectious SAA and ruptured aneurysms, even though some cases were small in size. The most significant impact of the 2020 guideline revision was the increase in the size threshold for treatment intervention in RAA and SAA from 2 to 3 cm. As a result, the aneurysms that did not meet the treatment criteria included 10 RAAs, 8 SAAs, and 2 HAAs. All the cases were considered ineligible solely due to the size criteria. In the largest observational study on RAAs, reported by Klausner et al., 865 RAAs were followed for an average of 29 months, with only 3 cases of rupture, all of which occurred in aneurysms larger than 3 cm. This accounted for 18% of all RAAs larger than 3 cm.^8)^ Another study of 338 RAAs reported that during an average follow-up of 24 months, there were no interventions for aneurysms smaller than 25 mm, and no ruptures were observed during follow-up. The study concluded that RAAs are more benign and less prone to rupture than previously thought, and that the 3 cm threshold recommended in the SVS guidelines is deemed appropriate.^11)^

On the other hand, the treatment indication for aneurysms larger than 3 cm in both renal hilar branch aneurysms and renal artery trunk aneurysms may need to be reconsidered. In cases of aneurysms located near the renal hilum, the use of stent grafts has made minimally invasive treatment possible. However, if a 2-cm aneurysm is monitored without intervention and later enlarges, leading to renal hilum expansion, the opportunity for stent graft treatment may be missed, necessitating OS. According to Venturini, a study involving 70 cases treated with embolization and 30 cases treated with stent grafts for VAAs reported that 6 of the 14 RAAs were treated with stent grafts. Stent grafts showed slightly better efficacy than embolization over an average follow-up period of 20 months, and mid-term patency rates were favorable.^12)^ Furthermore, a retrospective comparison of 41 cases treated with OS and 45 cases treated with EVT for RAAs reported that although covered stents were used in only 4 cases, EVT led to shorter ICU stays, shorter hospitalization periods, and lower estimated blood loss compared to OS.^13)^ Therefore, for high-risk patients who are unsuitable for general anesthesia, EVT may be worth considering for aneurysms in the 2-cm range.

Regarding the treatment of VAAs, the SVS guidelines recommend revascularization for RAAs when feasible. In contrast, for SAAs, the preservation or revascularization of the splenic artery is deemed unnecessary because of the collateral flow from the short gastric arteries. At our department, we considered stent graft placement for some cases of SAA; however, due to the lack of insurance coverage and the severe tortuosity of the splenic artery in all cases, we found it anatomically unsuitable and opted for coil embolization. Indeed, technical failures have been reported due to the inability to deliver the stent graft in cases of severe tortuosity.^14)^ Among the 12 cases in which we performed coil embolization, 4 cases developed partial splenic infarction. Based on this, we believe that, unless the patient is pregnant or the aneurysm shows significant enlargement, treatment of SAAs of 2 cm in diameter is unnecessary without careful consideration.

By retrospectively analyzing our cases using the 2020 SVS guidelines, we were able to clarify how many cases might have been over-indicated for treatment in the past— cases that, under current guidelines, would have been managed with observation instead of intervention. Historically, the treatment threshold for VAAs had been 2 cm, but with the updated 3-cm threshold for renal and SAAs, we now recognize that some patients underwent treatment when conservative management might have been sufficient. As expected, surgery/EVT imposes physical and psychological burdens on patients and carries the risk of postoperative complications. Furthermore, optimal treatment indications must always be considered from the perspective of efficient healthcare resource utilization.

The 1st limitation of this study is that, ideally, VAAs should be analyzed separately based on their specific locations. However, due to the limited number of cases in our single-center study, we retrospectively reviewed all cases collectively under the category of VAAs.

The 2nd limitation is that this study only includes treated cases. We did not examine cases where surgery/EVT was deferred for various reasons or the natural course of incidentally discovered small aneurysms (≤2 cm). As a result, we could not determine the total number of VAAs diagnosed during the study period or the proportion of cases that ultimately underwent treatment.

The significance of this study lies primarily in the finding that, when applying the size threshold of the 2020 SVS guidelines, nearly half of the cases were considered over-indicated for treatment. Additionally, regarding the treatment strategy outlined in the 2020 SVS guidelines—which states that “except for RAAs, EVT should be the 1st-line treatment, with OS reserved for cases where distal organ perfusion cannot be ensured”—our institution has generally followed a similar approach.

Considering that no prospective clinical studies have been conducted in Japan under this treatment strategy, the fact that our patients demonstrated acceptable early postoperative outcomes and a relatively long median follow-up period of 101 months suggests that treatment according to the 2020 SVS guidelines is appropriate. This represents the 2nd major significance of our study.

However, whether expanding the treatment threshold from 2 to 3 cm is truly appropriate remains uncertain because there are no prospective randomized studies evaluating the appropriateness of expanding the treatment indication from 2 to 3 cm, and further studies regarding the treatment threshold are necessary to validate this change.

## Conclusion

A retrospective comparison of the surgical indications in the 2020 SVS guidelines with the VAA treatment cases treated at our department showed a significant reduction in the number of eligible cases for treatment in RAAs and SAAs. This reduction was primarily due to the change in the size threshold for treatment intervention from 2 to 3 cm. However, as stated in the guidelines, aneurysms with a tendency for enlargement, as well as cases involving young women, ruptures, symptomatic aneurysms, or pseudoaneurysms, should still be considered for treatment even if the aneurysm size is below the recommended threshold.

## Declarations

### Disclosure statement

All authors have no conflicts of interest.

### Author contributions

Study conception: NI, KI, and NU

Data collection: NI, KI, and KK

Writing: NI and NU

Critical review and revision: all authors

Final approval of the article: all authors

Accountability for all aspects of the work: all authors.
